# Dysregulation of Ambient Glutamate and Glutamate Receptors in Epilepsy: An Astrocytic Perspective

**DOI:** 10.3389/fneur.2021.652159

**Published:** 2021-03-22

**Authors:** Oscar B. Alcoreza, Dipan C. Patel, Bhanu P. Tewari, Harald Sontheimer

**Affiliations:** ^1^Glial Biology in Health, Disease, and Cancer Center, Fralin Biomedical Research Institute, Virginia Tech Carilion, Roanoke, VA, United States; ^2^School of Medicine, Virginia Tech Carilion, Roanoke, VA, United States; ^3^Translational Biology, Medicine and Health, Virginia Tech, Blacksburg, VA, United States

**Keywords:** glutamate homeostasis, System x_c_^-^, epilepsy, astroglia, metabotrophic glutamate receptor, NMDAR

## Abstract

Given the important functions that glutamate serves in excitatory neurotransmission, understanding the regulation of glutamate in physiological and pathological states is critical to devising novel therapies to treat epilepsy. Exclusive expression of pyruvate carboxylase and glutamine synthetase in astrocytes positions astrocytes as essential regulators of glutamate in the central nervous system (CNS). Additionally, astrocytes can significantly alter the volume of the extracellular space (ECS) in the CNS due to their expression of the bi-directional water channel, aquaporin-4, which are enriched at perivascular endfeet. Rapid ECS shrinkage has been observed following epileptiform activity and can inherently concentrate ions and neurotransmitters including glutamate. This review highlights our emerging knowledge on the various potential contributions of astrocytes to epilepsy, particularly supporting the notion that astrocytes may be involved in seizure initiation via failure of homeostatic responses that lead to increased ambient glutamate. We also review the mechanisms whereby ambient glutamate can influence neuronal excitability, including via generation of the glutamate receptor subunit GluN2B-mediated slow inward currents, as well as indirectly affect neuronal excitability via actions on metabotropic glutamate receptors that can potentiate GluN2B currents and influence neuronal glutamate release probabilities. Additionally, we discuss evidence for upregulation of System ${\text{x}}_{\text{c}}^{-}$, a cystine/glutamate antiporter expressed on astrocytes, in epileptic tissue and changes in expression patterns of glutamate receptors.

## Introduction

The critical roles of astrocytes in supporting the healthy development and maintenance of a mature brain has been firmly established in the last few decades. It is well-appreciated that an imbalance between excitatory and inhibitory neurotransmission causes hyperexcitability in neuronal circuitry and underlies the processes of ictogenesis and epileptogenesis. Given the important functions that glutamate serves in excitatory neurotransmission, understanding the mechanisms regulating glutamatergic drive under physiological and pathological states provides critical insights into devising strategies to maintain glutamate homeostasis. Since 1-in-3 epilepsy patients are pharmacoresistant to currently available antiseizure drugs, that mainly target neuronal mechanisms (1, 2), elucidating the astrocytic processes involved in seizure generation and epileptogenesis in detail may help identify new targets to treat intractable forms of epilepsy.

**Figure 1:**
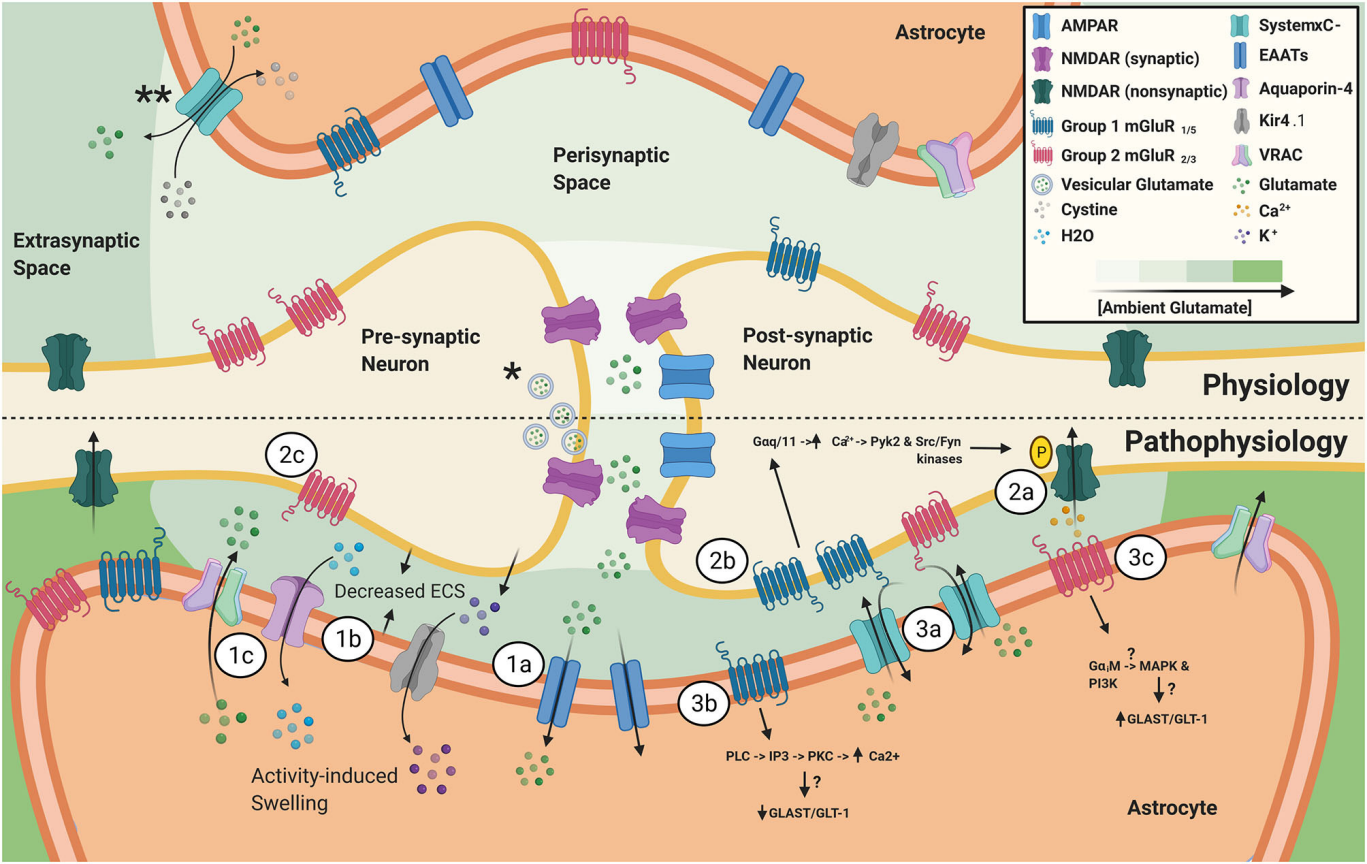
*Vesicular glutamate release during action potentials is the primary source of synaptic glutamate. **SXC, primarily expressed on astrocytes, is a major source of ambient, extrasynaptic glutamate. Ambient glutamate concentration around the synapse, after EAAT activity, follows a gradient with the lowest level in the synaptic cleft to the highest in the extrasynaptic compartment. **(1)** Astrocyte homeostatic responses to increased activity from hyperexcitable neurons. **(1a)** Increased vesicular glutamate release from hyperexcitable neurons leads to increased astrocytic EAAT activity. **(1b)** Elevated neuronal activity also causes release of K^+^, in attempts to maintain homeostatic neuronal resting membrane potential. Next, astrocytic buffering of extracellular K^+^ through elevated Kir4.1 activity, which is accompanied by increased H20 uptake through aquaporin-4, ultimately results in activity-induced astrocytic swelling and reduction in ECS. **(1c)** Astrocytic swelling leads to activation of VRAC and release of glutamate and other gliotransmitters into the ECS. **(2)** Pathophysiological effects of increased activity and changes in expression of neuronal extrasynaptic glutamate receptors. **(2a)** Activation of N2B-containing NMDARs leads to the generation of slow, depolarizing currents. **(2b)** Elevated expression and activity of group 1 mGluRs in epilepsy has been linked to increased NMDAR-mediated currents via a mechanism involving Ca^2+^-calmodulin dependent tyrosine phosphorylation of NMDAR subunits NR2A/B. **(2c)** Presynaptic group 2 mGluRs have been shown to inhibit glutamate and GABA release. Tissue from epileptic patients and animal models have revealed decreased mGluR_2/3_ expression, which can contribute to a pro-epileptic brain state. (3) Changes in astrocytic glutamatergic protein expression in epilepsy. **(3a)** SXC expression has been found to be elevated in human epileptic tissue, as well as various epilepsy animal models. SXC activity leads to the release of glutamate from astrocytes. **(3b)** Animal models of epilepsy have revealed that persistent upregulation of astrocytic mGluR_5_ was a reliable indicator of epileptogenesis. mGluR_5_ activation leads to altered GLAST/GLT-1 expression and induces NR2B-dependent NMDAR mediated neuronal currents. **(3c)** Upregulation of mGluR_3_ has been reported in epilepsy animal models and experimental activation of group 2 mGluRs in cultured astrocytes was shown to upregulate GLAST/GLT-1 expression, suggesting that a balance of group 1 and group 2 mGluRs on astrocytes is important in maintaining homeostatic extracellular glutamate.

This review serves to highlight the emerging dynamic processes that astrocytes undergo in epilepsy, in support of the notion that astrocytes play a critical role in seizure generation via homeostatic responses such as the increase in ambient glutamate through a reduction in extracellular space (ECS) following activity-dependent astrocytic potassium uptake and buffering (Figure 1-1). Importantly, we also review the mechanisms in which increased ambient glutamate can directly influence neuronal excitability, via generation of the glutamate receptor subunit GluN2B-mediated slow inward currents (SICs), as well as indirectly affect neuronal excitability via actions on metabotropic glutamate receptors (mGluRs) that can potentiate GluN2B currents, alter extracellular glutamatergic clearance, and influence neuronal glutamate release probabilities (Figure 1-2). Additionally, evidence of upregulation of System ${\text{x}}_{\text{c}}^{-}$ (SXC), a cystine/glutamate antiporter expressed on astrocytes, in epileptic tissue and changes in expression patterns of glutamate receptors have provided insights in how astrocytic dysregulation can contribute to seizure generation and epileptogenesis (Figure 1-3).

## Glutamate Synthesis, Release, and Reuptake

Before discussing the mechanisms whereby astrocytes regulate glutamatergic neurotransmission, it is important to understand the fundamental functions astrocytes play in CNS glutamate homeostasis. Glutamate or glutamic acid is a ubiquitous biological molecule serving multiple functions—mainly as an amino acid for protein synthesis, as a principal excitatory neurotransmitter in the mammalian CNS and as a source of energy (3). The use of an abundant amino acid as a neurotransmitter poses a significant challenge in the regulation of available glutamate in the CNS, since excess extracellular glutamate is highly neurotoxic. The evolution of two remarkable features of the biological system—the blood-brain barrier (BBB) and the astrocytic specializations—has solved the problem of potential “spillover” of peripheral glutamate into the CNS as a neurotransmitter. Interestingly, in both cases astrocytes are involved to the extent that glial dysfunctions can alter glutamate homeostasis and cause excitotoxicity and neuronal hyperexcitability.

The BBB is impermeable to glutamate (4), which is critical in preventing the flooding of the CNS by peripheral glutamate within the vasculature. However, this system also necessitates autonomous turnover of glutamate in the brain. The role of astrocytes in *de novo* synthesis of glutamate in the brain is well-established in literature (5). Astrocytes specializations, namely exclusive expression of pyruvate carboxylase and glutamine synthetase, allow them to serve critical functions in CNS glutamate homeostasis (6, 7). Since pyruvate carboxylase is essential for the synthesis of oxaloacetate that is subsequently utilized in the synthesis of α-ketoglutarate and glutamate, astrocytes are the only cell type in the brain capable of *de novo* synthesis of glutamate by the oxidative metabolism of glucose. Although one study recently identified another source of glutamate in the brain where glutamate is synthesized by neurons from blood urocanic acid (8), more studies are required to validate it as well as to estimate the relative contribution of neurons in total glutamate synthesis in the brain. Glutamate is subsequently amidated by glutamine synthetase into glutamine, which enters the glutamate-glutamine cycle between astrocytes and neurons.

After release into the synaptic cleft, excessive glutamate must be cleared quickly to prevent neurotoxicity. There are no extracellular enzymes that can neutralize glutamate (9), however, astrocytes can rapidly clear glutamate from the synaptic clefts via astrocytic processes that completely enclose many glutamatergic synapses. These astrocytic processes also express highly efficient excitatory amino acid transporters (10) (EAATs) that take up 80% of extracellular glutamate in the CNS (5) (Figure 1-1a). Once inside the astrocyte, it is estimated that ~85% of glutamate is converted into glutamine and returned to neurons, while the remaining ~15% is metabolized to α-ketoglutarate and further oxidized through the tricyclic acid cycle for energy production. This helps to cover the energy costs of glutamate handling as both pyruvate carboxylase and glutamine synthetase catalyze energy-dependent reactions (3). Insights into the synthesis and regulation of glutamate in the CNS serve to highlight the role that astrocytes play in health and to set up the potential consequences of disrupting astrocytic glutamate homeostasis in the pathology of epilepsy.

## Astrocytic Regulation of Ambient Glutamate via Volumetric Shifts

Several recent reviews have emphasized the relationship between cerebral edema, astrocytic swelling, and epilepsy, due to a strong link between the volume reduction of the ECS hyperexcitability (11, 12). ECS is a narrow space between CNS cells that serves as a reservoir of water, ions, and signaling molecules to maintain ionic and water homeostasis. Interestingly, ECS reduction is one of the common mechanisms of generating hyperexcitability in several forms of epilepsies and astrocytes play key role in ECS volume regulation.

Astrocytes can significantly alter the volume of the CNS ECS due to their expression of the bi-directional water channel, aquaporin-4 (AQP4), which is enriched at perivascular endfeet (13). Neuronal activity-dependent astrocytic swelling, following potassium uptake and buffering, is an important mediator in the reduction of ECS (Figure 1-1b). Indeed, measurements of the ECS before and during bath application of picrotoxin, a GABA_A_ receptor antagonist, to induce epileptiform activity revealed that brief epileptiform discharges rapidly decreased the median ECS width, measured as the width of interstitial space separating neural structures, by over 50% (14). Rapid ECS shrinkage can inherently concentrate ions and neurotransmitters including glutamate, explaining elevated extracellular glutamate during seizures in epilepsy patients (15). ECS shrinkage due to astrocyte swelling in hypoosmolar conditions has been shown to be sufficient in evoking large excitatory slow inward currents (SICs) in neurons (16). Conversely, studies in AQP4-knockout animals have revealed that these mice have larger ECS, slower potassium kinetics and are more resistant to seizure generation using pentylenetetrazole (PTZ) (17–19), a GABA_A_ receptor antagonist.

Another way astrocytes regulate water homeostasis and consequently ECS volume is the volume-regulated anion channel (VRAC), which are typically activated through hypotonicity-induced cell swelling (20, 21). VRAC belongs to the leucine-rich repeat-containing 8 (LRRC8) family of proteins (22, 23) and consists of a heterogenous mix of LRRC8 proteins of which LRRC8A, also known as Swell1, is an essential subunit (24). Upon astrocytic swelling, VRAC is activated and allows the release of chloride ions and other osmolytes, including glutamate (Figure 1-1c). This in turn generates an osmotic gradient that drives water out of the astrocyte. Using astrocyte specific *Swell1* knockout mice and VRAC inhibitors, recent studies have identified that VRAC-mediated glutamate release can modulate NMDAR-mediated tonic currents, as well as affect neuronal excitatory vesicle release probabilities (25, 26).

These processes support the hypothesis that impairment in astrocyte functions may play a critical role in initial seizure generation by increasing physiologically relevant ambient glutamate through (1) a reduction of ECS, following astrocytic potassium uptake and buffering, (2) glutamate release through VRAC or (3) a dysregulated state combining both mechanisms.

## Astrocytic Regulation of Ambient Glutamate via SXC

Studies investigating the physiological role(s) of ambient glutamate [reviewed in (27)] have identified several diverse functions such as involvement in sleep-wakefulness cycles, synaptic plasticity, and the ability to influence neuronal resting membrane potentials via induction of NMDAR-mediated inward currents. However, the processes that maintain and regulate levels of ambient glutamate continue to be elucidated (27). Another important source of ambient, extracellular glutamate is via the sodium independent activity of the cystine/glutamate antiporter SXC. SXC is a covalently coupled heterodimeric protein complex comprised of the 4F2 heavy chain, *SLC3A2*, linked to the cystine/glutamate exchanger (xCT), *SLC7A11*. Notably, while xCT is abundantly expressed in cells throughout the body, examination of SXC knockout mice (xCT^−/−^) mice revealed that in the CNS, xCT is exclusively expressed in astrocytes, and absent in neurons, oligodendrocytes, and microglia (28). xCT plays a major role in the modulation of ambient extracellular glutamate as pharmacological inhibition of SXC, using (S)-4-carboxyphenylglycine (S-4-CPG), resulted in a 60% reduction in extrasynaptic glutamate in the nucleus accumbens (29). Although S-4-CPG is known to inhibit type 1 mGluRs, this study found that use of the type 1 mGluR antagonist (RS)-1-aminoindan-1,5-dicarboxylic acid (AIDA) did not result in changes in ambient glutamate. This finding was confirmed recently using antisense xCT which similarly decreased extracellular glutamate in the nucleus accumbens (30). Additionally, xCT^−/−^ mice have around 30% less hippocampal ambient glutamate (31).

In tumor-associated epilepsy, overexpression of SXC in glioblastomas and the corresponding increase in peritumoral glutamate levels have been directly associated with the development of seizures and poor patient survival (32–34). These studies also found that inhibition of SXC via sulfasalazine (SAS), an FDA approved drug used to treat inflammatory bowel disease, reduced epileptiform activity and seizure frequency both *in vitro* and *in vivo* in glioblastoma models. In addition to tumor-associated epilepsy, resected human epileptic tissue from patients with temporal lobe epilepsy (TLE) also had an elevated expression of SXC (35) (Figure 1-3a). We have also shown recently that SAS could significantly decrease the frequency and/or amplitude of evoked excitatory postsynaptic currents in multiple *in vitro* models of hyperexcitability (36). Furthermore, co-application of SAS with topiramate, an FDA-approved anti-seizure drug, further decreased epileptiform activity synergistically compared to topiramate alone.

The role of SXC in neuronal hyperactivity via ambient glutamate regulation is further supported by recent *in vivo* studies on xCT^−/−^ mice showing delayed epileptogenesis and reduced seizures in the self-sustained status epilepticus (SSSE) and pilocarpine induced status epilepticus models (37). Additionally, this study found decreased micro- and astrogliosis in xCT^−/−^ mice after SSSE. In contrast, wildtype mice that underwent pilocarpine-induced status epilepticus had significantly increased xCT expression during latent phase of epileptogenesis. Using another strain of SXC knockout mice, which have a spontaneous deletion in the xCT gene in subtle gray mice (xCT^sut/sut^), it was found that xCT^sut/sut^ mice were significantly resistant to epileptic kindling compared to wildtype mice. Additionally, western blot analysis of plasma membrane proteins found that cortical, but not hippocampal, surface GluA1 expression was significantly decreased in xCT^sut/sut^ mice (38).

In contrast to the above studies that show a pivotal role of SXC in hyperexcitability, a recent study using the Theiler's Murine Encephalomyelitis Virus (TMEV) model of viral-induced epilepsy found that xCT^−/−^ mice were not protected against this form of epilepsy and had similar number and severity of behavioral seizures (39). The lack of difference in TMEV-induced seizures between xCT^−/−^ and WT mice can be possibly explained by the fact that the proinflammatory cytokines, tumor necrosis factor-α and interleukin-6, are known as the major drivers of hyperexcitability and seizures in this epilepsy model (40, 41). Taken together, these above cited studies establish SXC as a potential astrocytic drug target. Clearly astrocytes have the capabilities to modulate ambient extracellular glutamate, yet whether astrocytic SXC exerts a direct, pro-epileptic effect by modulating ambient glutamate in acquired epilepsies requires further study.

## Mechanisms of Astrocyte-Derived Glutamate Contributing to Neuronal Hyperexcitability

So far, we have discussed that astrocytes not only prevent excitotoxic glutamate accumulation in the ECS, but also serve as a source of extracellular glutamate under specific conditions. However, important questions remain. How might astroglial glutamate contribute to neuronal activity, and is it sufficient to trigger neuronal hyperactivity? Additionally, how might different types of glutamate receptors at tripartite synapses and extrasynaptic spaces respond to fluctuations in ambient glutamate?

The notion of glial-neuronal crosstalk via gliotransmission has been extensively studied in the last two decades. Regardless of the mechanisms, a wealth of evidence has demonstrated that astrocytes can sense neuronal activity and respond through the release of gliotransmitters including ATP, D-serine, and glutamate (42, 43). Research into the origin and consequences of neuronal SICs revealed that these currents are generated via astrocyte-derived glutamate acting on extrasynaptic GluN2B subunit-containing N-methyl D-aspartate type of glutamate receptors (44, 45) (NMDARs) (Figure 1-2a). Extrasynaptic NMDARs are thought to have increased GluN2B-containing heterodimers, suggesting a spatially specific function. Indeed, GluN2B-containing NMDARs have a higher affinity for glutamate compared to GluN2A-containing NMDARs (46), which may facilitate sensing glutamate in the extrasynaptic space that has far lower extracellular glutamate compared to an active synapse.

SICs can be synchronized in multiple neurons over 100 microns apart in the hippocampus, raising the possibility of astrocytic synchronization of neuronal hyperactivity in epilepsy. Interestingly, blockade of glutamate uptake and exocytotic glutamate release increased the frequency and amplitude of these NMDAR-mediated SICs, suggesting that increases in ambient glutamate could play a physiologically relevant role (45). A study investigating the actions of extrasynaptic glutamate determined that SXC-mediated glutamate release preferentially activates extrasynaptic GluN2B-containing NMDARs (47). Similarly, another study determined that the glutamate responsible for generating SICs occur independently from exocytotic Ca^2+^-dependent glutamate release (48). Notably, a paper examining the sensitivities of glutamate receptors to glutamate predicted the percentage of glutamate receptors that would remain activated at increasing levels of ambient extracellular glutamate (49). This paper succinctly demonstrates that NMDARs and mGluRs would be preferentially activated by small, local fluctuations in astrocytic glutamate release as they are activated by glutamate concentrations around 1–30 μM, while α-amino-3-hydroxy-5-methyl-4-isoxazolepropionic acid type of glutamate receptors (AMPARs) are only activated in the presence of 100–3,000 μM of glutamate. Therefore, it is evident that astrocytes possess the molecular machinery necessary to significantly alter the concentration of extrasynaptic ambient glutamate, which can act on NMDARs to induce neuronal SICs that can contribute to hyperexcitability.

## Metabotropic Glutamate Receptors and Epilepsy

Metabotropic glutamate receptors (mGluRs), which are coupled to G protein-coupled pathways and G protein-independent pathways, are another class of glutamatergic receptors whose dysregulation have been implicated in the pathology of epilepsy. Unlike ionotropic glutamate receptors, mGluR activation can trigger long-term changes in cellular signaling by regulating the expressions of various homeostatic and glutamatergic proteins in neurons and glial cells (50). Currently, eight mGluR subtypes have been described that fit into three subgroups (51, 52). Group 1 mGluRs consists of mGluR1 and mGluR5 and are coupled to Gα_q/11_, which stimulates the release of Ca^2+^ from intracellular stores upon activation. Group 1 mGluRs are thought to be distributed primarily on post-synaptic neurons, in the perisynaptic zone (53, 54). Using the kainic acid (KA) rat model of TLE, one study has reported upregulation of neuronal mGluR1 in rodent hippocampi, in addition to, mGluR1 upregulation in human TLE tissue (55). Importantly, neuronal mGluR1 activation was shown to potentiate NMDAR-mediated currents via a mechanism involving Ca^2+^, calmodulin and Src-dependent activation of proline-rich tyrosine kinase leading to tyrosine phosphorylation of NMDAR subunits GluN2A/B (56) (Figure 1-2b). A study investigating the efficacy of mGluR1 inhibition in epilepsy found that mGluR1 inhibition decreased PTZ-induced seizures, and this effect could be prevented by adding mGluR1 agonists (57).

mGluR5 is also expressed on cortical and hippocampal astrocytes, primarily during development (58), however, the reappearance of mGluR5 expression has been observed in astrocytes of specific epilepsy animal models, and human epileptic tissue (59–61). Intriguingly, mice with persistent astrocytic mGluR5 expression during the latent period reliably went on to develop epilepsy, whereas mice with only transient mGluR5 expression did not (59) (Figure 1-3b). Additionally, epileptic mice with astrocytic mGluR5 knocked out displayed lowered glutamate uptake kinetics during high-frequency stimulation compared to epileptic wildtype mice. Downstream effects of astrocytic mGluR5 activation include increased Ca^2+^-dependent, GluN2B-subunit containing NMDAR-mediated neuronal currents (61). *In vivo* loading of BAPTA-AM, a Ca^2+^chelator, selectively into astrocytes was found to be neuroprotective and significantly reduced the amount of Fluoro-Jade B labeling of dying neurons. Together, these findings suggest that reappearance of astrocytic mGluR5 may be a useful biomarker for active epileptogenesis, as well as a contributor to epileptogenesis by potentiating GluN2B-mediated inward neuronal currents.

Group 2 mGluRs consist of mGluR2 and mGluR3, while Group 3 mGluRs consist of mGluR4, mGluR6, mGluR7, and mGluR8. Both Group 2 and Group 3 inhibit adenylyl cyclase activity through Gα_i_ activation and are thought to be largely distributed on pre-synaptic neurons, where they act to inhibit glutamate and GABA release (62, 63). Additionally, mGluR3 has been reported to be expressed on post-synaptic neurons and astrocytes (64–66). Using the pilocarpine TLE animal model, studies have shown that neuronal mGluR2/3 expression in the hippocampus (67, 68) and cortex (69) are decreased in epilepsy. Additionally, epileptic tissue from patients with TLE also have decreased mGluR2/3 expression (68) (Figure 1-2c). As pre-synaptic Group 2 mGluRs are thought to inhibit glutamate release, down-regulation of these receptors could promote glutamate release and neuronal hyperexcitability. Indeed, studies using Group 2 mGluR agonists have been shown to be neuroprotective in the models of absence epilepsy and the epilepsy models induced through amygdala kindling (57, 70). Conversely, Group 2 mGluR antagonists were shown to be pro-epileptic in an absence epilepsy model (71). Notably, these researchers also found that a mGluR1 potentiator, 9H-xanthene-9-carboxylic acid(4-trifluoromethyl-oxazol-2-yl)amide (SYN119), played a protective role against spike and wave discharges in a rat model of absence epilepsy (72).

One study investigating the localization and changes in mGluR3 and mGluR5 expression after hippocampal injury via KA-induced seizures found that mGluR3 mRNA was exclusively upregulated in astrocytes and oligodendrocytes (Figure 1-3c). Additionally, GFAP-positive astrocytes were found to be persistently upregulated from 2 days to 12 weeks post hippocampal injury (73). Although mGluR5 mRNA was not found to be upregulated in astrocytes, other studies have reported upregulated mGluR5 protein levels in animal models of epilepsy (74, 75). To understand how changes in astrocytic mGluR3 or mGluR5 activity may affect seizure generation, one study looked at the effects of adding Group I and Group II modulators to cultured astrocytes (76). This paper found that astrocytic Group 1 mGluR activation, via the use of (S)-3,5-dihydroxyphenylglycine [(S)-3,5-DHPG], led to decreased expression of glutamate transporters (GLAST and GLT-1), and that this effect could be antagonized using a selective mGluR5 antagonist, MPEP. Conversely, astrocytic Group II mGluR activation, using DCG-IV, resulted in upregulation of GLAST and GLT-1 expression, and this effect could be abolished using the Group II antagonist EGLU. As mGluR2 has not been found to be expressed by astrocytes, the authors concluded that the effects of Group II modulation in these experiments was attributable to astrocytic mGluR3 activity.

Questions remain and more work certainly needs to be done to further elucidate the role of mGluRs in epilepsy, however, it appears evident that mGluR expression and activity are significantly altered both in animal models and human epileptic tissue in ways that contribute to hyperexcitability and epileptogenesis. Although ionotropic and metabotropic glutamate receptors differ fundamentally in their structure and downstream effectors, NMDARs and mGluRs share the ability to sense changes in ambient glutamate and potentiate neuronal inward currents.

## Discussion

Supporting the notion that astrocytes are critically involved in the initiation of seizures through dysregulation of ambient glutamate, a recent *in silico* study found that increased ambient glutamate, either through increased astrocytic glutamate release or decreased uptake, was sufficient to initiate synchronous epileptiform-like discharges from neurons (77). Additionally, the use of transparent zebrafish combined with two-photon calcium imaging and local field potential recordings allowed investigators to monitor the activity and connectivity of thousands of neurons and glia revealing new insights into the role glia may play in seizure generation (78). Using PTZ, a GABAaR antagonist, to initiate seizures investigators found that pre-ictal neuronal activity did not significantly increase in synchrony, however, radial glia exhibited a significant increase in synchrony during the pre-ictal period followed by neuronal bursts. These findings support the idea that the local, pro-epileptic glial responses presented throughout this review can culminate in network-level events that can ultimately lead to neuronal hyperexcitability synchronization and seizure initiation.

Knowing that current anti-seizure therapeutics are ineffective for 1-in-3 people living with epilepsy (1, 2), elucidating the astrocytic processes involved in epileptogenesis may help identify new therapeutic targets that offer relief to patients with intractable epilepsy. Table 1 summarizes current FDA-approved and investigational pharmacotherapies targeting glutamate signaling for epilepsy. In summary, astrocytes exclusively possess the enzymatic activity to generate *de novo* glutamate in the CNS, as well as the molecular machinery to determine ambient glutamate through (1) a reduction of ECS, (2) glutamate release through SXC or VRAC or (3) a dysregulated state combining these mechanisms. Importantly, ambient glutamate can directly influence neuronal excitability, via generation of GluN2B-mediated SICs, as well as indirectly affect neuronal excitability via actions on mGluRs that can potentiate GluN2B-mediated currents, alter extracellular glutamatergic clearance, and influence neuronal glutamate release probabilities. Although inherent challenges exist in ubiquitously targeting glutamatergic mechanisms in the CNS, it appears clear that astrocytes are uniquely positioned to act as a master regulator of glutamate in health, and when dysregulated, can mediate pro-epileptic changes that warrant further investigation in hopes of unveiling novel therapeutic targets.

**Table 1:** Pharmacotherapies targeting glutamate signaling for epilepsy.

**Drug**	**Brief mode of action**	**Effects on glutamatergic system**	**Effects on seizures**	**Developmental Stage**	**Reference**
Perampanel	Noncompetitive selective AMPAR antagonist	↓ AMPAR-mediated fast excitatory neurotransmission	↓ focal and generalized tonic-clonic seizures	FDA-approved in 2012	(79)
Topiramate	AMPAR/KAR inhibitor; multiple other mechanisms	↓ excitatory neurotransmission	↓ focal and generalized convulsive seizures	FDA-approved in 1995	(80)
Felbamate	NMDAR inhibitor; multiple other mechanisms	↓ excitatory neurotransmission	↓ focal and generalized convulsive seizures	FDA-approved in 1993	(81)
Ketamine	NMDAR antagonist	↓ NMDAR-mediated excitatory neurotransmission; potentially neuroprotective	efficacious against refractory status epilepticus	Under clinical trial	(82)
Gabapentinoids (Gabapentin, Pregabalin)	Blocker of α2δ subunit of voltage-gated Ca^2+^ channel	↓ release of glutamate	↓ focal seizures (gabapentin, pregabalin)	FDA-approved (Gabapentin, 1993; Pregabalin, 2004)	(83)
		↓ excitatory synaptogenesis	↓ generalized convulsive seizures (gabapentin)		(80)
		inhibits surface trafficking and synaptic targeting of NMDAR			
Levetiracetam	Synaptic vesicle glycoprotein 2A (SV2A) modulator	↓ release of glutamate, ↑ synaptic depression	↓ focal and generalized tonic-clonic seizures; potentially antiepileptogenic	FDA-approved in 2000	(84, 85)
Brivaracetam	SV2A modulator (more selective than levetiracetam)	↓ release of glutamate, ↑ synaptic depression	↓ focal and generalized tonic-clonic seizures	FDA-approved in 2016	(84, 85)
17AAG	HSP90β inhibitor	inhibits internalization and proteosomal degradation of GLT-1	↓ seizures in intrahippocampal kainate model of epilepsy	Investigational	(86)
		↑ glutamate clearance from ECS			
Ceftriaxone	GLT-1 transcriptional activator	↑ glutamate clearance from ECS	↓ frequency and duration of post-traumatic seizures	Investigational	(87)
		↓ excitotoxic loss of inhibitory interneurons			(88)
		↑ intracellular glutathione and ↓ oxidative stress			(89)
Sulfasalazine	System ${\text{x}}_{\text{c}}^{-}$ transporter inhibitor	↓ extracellular level of glutamate	↓ seizures in a murine model of tumor-associated epilepsy	Investigational	(32)
		↑ intracellular glutathione and ↓ oxidative stress			
LY367385, LY339840	mGluR1 antagonist	↓ excitatory neurotransmission	Potent anticonvulsant activity in animal models of seizures	Investigational	(90, 91)
		↓ glutamate release from presynaptic terminals and perisynaptic astrocytic processes			(92)
MPEP	mGluR5 negative allosteric modulator	↓ excitatory neurotransmission	Potent anticonvulsant activity in animal models of seizures	Investigational	(93, 94)
		↓ glutamate release from presynaptic terminals and perisynaptic astrocytic processes			(92)
S-4C3HPG	mGluR1 antagonist, mGluR2 agonist	↓ glutamate release and neurotransmission	Protects against audiogenic seizures in DBA/2 mice	Investigational	(95)
			Suppresses PTZ and DMCM-induced seizures		(96)
2R,4R-APDC	Group 2 mGluR agonist	↓ glutamate release and neurotransmission	Enhance seizure threshold in a rat model of amygdala kindling	Investigational	(97)
DCG-IV	Group 2 mGluR agonist	↓ glutamate release and neurotransmission	↓ kainate and amygdala kindling-induced seizures	Investigational	(98, 99)
JNJ-42153605, JNJ-40411813, JNJ-46356479	mGluR2 positive allosteric modulator	↓ glutamate release and neurotransmission	Anticonvulsant effect in the mouse 6-Hz and corneal kindling models	Investigational	(100, 101)
			Enhances antiseizure efficacy of levetiracetam		
LY404039	Group 2 mGluR agonist	↓ glutamate release and neurotransmission	Anticonvulsant effect in a model of 6 Hz psychomotor seizures	Investigational	(100)
			Enhances antiseizure efficacy of levetiracetam		

*17AAG, 17-allylamino-17-demethoxygeldanamycin; HSP90β, Heat shock protein 90β; MPEP, 2-methyl-6-phenylethynyl-pyridine; S-4C3HPG, S-4-carboxy-3-hydroxyphenylglycine; PTZ, pentylenetetrazol; DMCM, methyl-6,7-dimethoxy-4-ethyl-beta-carboline-3-carboxylate; 2R,4R-APDC, 2R,4R-1-aminocyclopentane dicarboxylic acid; DCG-IV, 2S,2′R,3′R-2-(2′,3′-dicarboxycyclopropyl)glycine*.

## Author Contributions

OA did an exhaustive literature search, generated a complete draft of the review, and prepared the figure. DP contributed written material and prepared Table 1. DP, BT, and HS also reviewed the literature, provided detailed comments and edits to the review, figure, and table. All authors contributed to the article and approved the submitted version.

## Conflict of Interest

The authors declare that the research was conducted in the absence of any commercial or financial relationships that could be construed as a potential conflict of interest.
